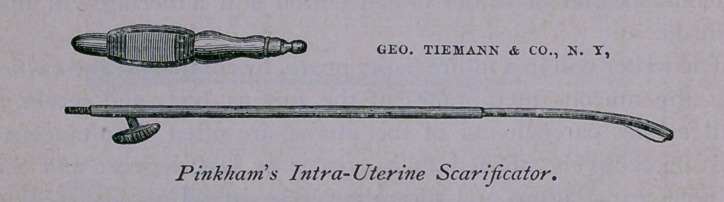# On a Few Instruments for the Practical Treatment of Uterine Disease

**Published:** 1871-05

**Authors:** Philip Adolphus

**Affiliations:** 169 Dearborn Street; Chicago


ARTICLE II.—*On a few Instruments for the Practical Treatment of Uterine Disease.* By PHILIP ADOLPHUS, M.D., Chicago.

(Third Paper—continued from March No.)

1st. Emmet's Applicator; used to remove secretions from the cervix and uterine cavity.

2d. Nott's Dilator.

3d. Pinkham's Intra-Uterine Scarificator.

Although these articles were written for the purpose of elucidating the topical treatment of uterine disease by instruments; yet constitutional treatment is in many cases requisite, and local treatment without it generally a failure. The treatment of chronic uterine disease has been brought into disrepute in some quarters by exclusive views, by advocating solely either mechanical appliances, topical applications, or general treatment.

To insure success, these three measures have to go hand in hand: Hygienic and medical treatment, with suitable applications to the parts affected, and the *simplest* mechanical appliances in skillful hands, will insure improvement in a class of cases proverbial for taxing the patience of the attendant and the faith of the patient.

There are a few principles to be observed in the use of instruments, and indeed in the treatment of these diseases generally, so indisputably sound, that they may be stated dogmatically.

1stly. *To endeavor to do the patient no injury with our treatment.* (Chomel).

2dly. *To be always hopeful.* "With rare exceptions, of which cancer is a prominent example, those who treat diseases of women may deal largely in hope, *and I feel my duty is not well done toward a patient, if she leaves me without believing that cure is certain, though it may be delayed. One great advantage of experience is that it teaches us to hope.* * * * * I have repeatedly seen such patients recover, if their courage can be kept up, so as to make them persevere, with more or less of active treatment, during one, two, or even three years."* "If a young physician called upon to take charge of a case of cancer should take up the most recent systematic works on gynæcology, to ascertain what he could do to arrest the progress of the disease, palliate symptoms, or to relieve suffering, I think the result would be a despondent impression as to any positive service he could render to his patient. Yet in few diseases are the resources of art so manifest and so conclusive as in this, for we can most certainly improve nutrition, relieve pain, secure sleep, and arrest the sanious offensive discharges and hemorrhage, and thus notably prolong life, and make it tolerable to the end. *I have often thought that the feeling on the part of the physician, that the case is hopeless, discourages the constant and assiduous care which may really be of great service to the patient.*"†

* Uterine Therapeutics, E. J. Tilt, 1869, fol. 7.

† Clinical Observations on Malignant Diseases of the Uterus. By Fordyce Barker, M.D. Am. Journal of Obstetrics, Nov. 1870, fol. 534.

It is therefore our manifest duty to hold out inducements for hope, proportionate to the nature and duration of the patient's disease. The rule which the writer follows in *all cases*, is to promise relief, sooner or later, and on being questioned when relief may be expected, he answers, that in many cases it will be realized in a shorter time than he would dare to promise; that patience, intelligent co-operation, and faith in his ability to relieve her, will render its accomplishment more speedy. To insure this faith on the part of the patient, and to fan the embers of hope brighter, he takes exact notes of the slightest of her symptoms, and at short periods, compares the state of the patient with these notes; it is then generally found that some of the symptoms are mitigated, or have entirely disappeared. In short, to insure success, it requires patience, faith in the powers of nature and one's own skill; these will inspire implicit confidence on the part of the patient. (Tilt).

Having introduced the Speculum (Nott's or Sims'), and probed the cavity of the womb, we are ready to apply topical treatment to the canal of the cervix or cavity of the organ, provided these parts are open for the escape of fluids, and the easy introduction of instruments. If not sufficiently pervious, sponge or sea-tangle tents are used, which will expand the cervix to almost any extent desired, but it is generally more convenient and expeditious to use metallic dilators when the parts are dilatable. The practice of Mackintosh, Priestly and Simpson, of dilating the cervix uteri for dismenorrhœa, has been adopted in the treatment of many other complaints; and it is necessary for the success and safety of any surgical procedure with the body of the womb. Both Kammerer* and Peaslee† use a series of sounds for the dilatation of the cervix, previously to applying intra-uterine medication; the first employs a set of four sounds, "which are graduated, that, by their successive introduction the uterine cavity can be dilated with the use of a moderate amount of force." (Kammerer). Peaslee "began to use, seven years ago, a set of steel dilators of his own devising, with the object of producing a far more rapid dilatation of the cervical canal than sponge-tents or laminaria can

* On the Treatment of Uterine Catarrh. Joseph Kammerer. Am. Journal of Obstetrics, Aug. 1869.

† N. Y. Med. Journal. Intra-Uterine Medication. July, 1870.

effect." They are five dilators, ranging from $\frac{1}{8}$ to $\frac{5}{16}$ of an inch in diameter, and he has found their use very satisfactory. J. C. Nott,* to effect the same object, uses a pair of forceps, which the writer prefers and which he recommends. "All tents are open to the objection that the dilatation produced by them is only temporary, and their repeated introduction, previous to every application into the uterine cavity, is too laborious" (Kammerer, oper. cit.), and the writer adds, in office practice impracticable. Although the dilatation produced with the metallic dilators is also of a transient nature, yet a few seconds will suffice to dilate the canal sufficiently for intra-uterine applications.

The writer prefers Nott's dilator, because it is an effective, powerful instrument, entirely within the control of the operator, when introduced into the cervix or urethra, where it may be expanded gradually, slowly or suddenly, being governed in its expansion entirely by the sensations of the patient. One introduction at each session, if dilatation is necessary at all, suffices in every case, and one instrument only is necessary; whereas, if Peaslee's and Kammerer's sounds are used, the smaller sound is followed by the larger, and if the direction of the cervical canal should be abnormal, difficulty and pain result.

*Nott's Dilating Forceps.*

They may be described as a pair of slightly curved forceps, with handles like scissors, which when closed have a diameter of one-sixth of an inch; at about two inches from their point the blades expand in a bulb-like protuberance, so that they can only pass that distance into the uterus. The mode of their introduction and application is as follows: place the patient in the usual position, introduce the speculum, fasten Sims' hook to the anterior lip of the cervix, ascertain the direction of the canal by means of the probe,

* Treatment of Endometritis by Uterine Injection. J. C. Nott. Am. Journal of Obstetrics, Nov. 1869, fol. 470.

follow it by Nott's dilator closed; expand the blades very gradually, slowly, carefully, requesting the patient to inform you when the pain cannot be borne. If so informed, desist at once, and after a few moments of intermission, again expand the blades. This will generally give the requisite permeability to the canal; if not, relinquish all further attempts for that day, apply a piece of cotton wool saturated with glycerine to cervix, a rectal pill of Morphia and Belladonna into the rectum, and postpone dilatation to the next day.

In cases where the canal of the cervix only is implicated, it is generally sufficiently open, even without dilatation, for the cleaning of the parts, and for the introduction of remedies by Emmet's applicator; but in cases where the mucous membrane or parenchyma of the body are affected, and it is desirable to clean the parts thoroughly by intra-uterine injection, (and it is always advisable to use Nott's catheter in these conditions of the uterus if complicated by flexions), then it becomes requisite to dilate the parts for the purpose of introducing the instrument with ease.

The dilator is harmless if properly handled, but the same precautions must be observed as with tents, viz., avoid its use in an irritable and sensitive condition of the parts. The aphorism of Byford "that a tender uterus is a diseased uterus" should always be remembered. A golden rule it is to pay strict attention to the *local* nervous symptoms, as well as to the *general* nervous manifestations of the *local* disease, before attacking it by disturbing appliances. This broad and catholic teaching of Hugh L. Hodge is an axiom in the treatment of pelvic disease of every description, which must never be ignored. The penalty of its neglect is danger of life to the patient, and loss of reputation to the practitioner. Therefore allay local irritation by local depletion, hot injections (Emmet), suppositories of glycerine to cervix (Sims), and suppositories of glycerine medicated (or simple), with extract of belladonna and extract of aconite into the uterine cavity, and suppositories into the rectum, as a preliminary step.

"Many words have been wasted, I think, on the danger of dilating the cervix uteri. Sponge tents, rapid abortions, Barnes' dilator, all act in the same way; and no one fears the effects of these, except the sponge, which is a putrifying, irritating material. The dangers of dilatation, whether gradual or sudden, are attributable to an inflamed or morbidly sensitive condition of the organ, and it is often important to allay irritation before the use of tents."*

It may be asked what kind of instrument should be used for the purpose of cleansing the canal of the cervix and the cavity of the womb, of accumulated secretions, which always render efficient treatment nugatory by their neutralizing influence, when chemically combined with the substances introduced. That instrument is the best, that, with a calibre permitting easy entrance into the cavity of the uterus, combines such flexibility as will permit it at *once* to assume in the hands of an experienced operator *any required shape.* It must be able to follow the most tortuous track in the canal of the womb, which had been traced by Sims' probe; and lastly, it should, when withdrawn from the uterine cavity, free itself of the fouled cotton wool, without the agency of the operator's hands, which would be soiled thereby.

Almost all the probes now in use have their faults. Budd's whalebone probe, *the best of all*, requires a preliminary moulding to any given position, by means of hot water, or heat, then a secondary hardening, which it acquires in cooling, before it is enabled to follow the channel traced out by Emmet's probe.

*Emmet's Applicator.*

We possess the *perfect instrument* in Emmet's applicator, made of annealed silver wire, flexible, yet with the requisite firmness, flattened at its upper extremity, and mounted on a light handle. It is almost the *fac-simile* of his improvement of Sims' probe; a little larger, slightly stouter, and flat at its extremity, without the bulb of the other instrument.

To this is added, for the purpose of perfecting it, a compressed coil of brass wire, five inches in length, hollow and flexible, resembling an enlarged watch spring, capped at each extremity

* J. C. Nott, op. cit.

by a hollowed bead of gutta percha, which bead is fashioned flat at one extremity of the coil and rounded at the other. This addition, devised by Sims, passes over the instrument as a slide, and acts, when the latter is armed with cotton wool and the coil is propelled towards its point, by pushing the roll of cotton off into the neck or cavity of the uterus.

This instrument may be advantageously used for cleaning the uterus in the following manner: Slip Sims' brass coil or slide over Emmet's applicator, arm it with cotton wool; (the patient is in the usual position, with speculum *in situ*, Sims' hook holding the anterior lip, Emmet's probe still in the cavity of the cervix or uterus, tracing out its direction,) now remove probe from uterus, examine its curve, and give the identical curve to the armed instrument, dip it in salt water (Peaslee), or very dilute muriatic acid water (Swann), for the purpose of dissolving the secretions of the cervix; again introduce Emmet's probe as a guide, and follow it by the armed applicator immediately. After cleaning the cavity, remove the instrument, and push the coil or slide towards the end of it, the fouled cotton still adhering will then drop off.

The writer will in a future paper prove, by competent authorities, that the mucous membranes of the fundus, body and cervix, as well as the parenchyma of the uterus, are often simultaneously and consecutively affected to a greater or less degree; and that erosions, excoriations and ulcerations co-exist. Congestion plays a very important part in these affections, it produces chronic uterine catarrh, this in turn induces lesions of the mucous membranes of the cervix; congestion farther developes chronic engorgement of the walls of the organ, as well as flexions, whilst flexions again cause congestions.

Here then we have the indications for local bloodletting; now leeching is too expensive, often painful, it causes annoyance to attendant and patient, and occupies much time. Scarifications cause no pain, if the instrument is in proper condition, and can be performed quickly and efficiently.

The congestions of the cervix may by Buttles' and Emmet's instruments be efficiently treated; but when the body and fundus of the uterus are implicated, and we wish to remove blood from these parts, some intra-uterine scarificator is essential.

To Miller, H. R. Storer and J. G. Pinkham, is the profession indebted for excellent intra-uterine scarificators. To Storer also belongs the distinction of having been the first to suggest intra-uterine scarification, which, when the profession generally practices it, will be acknowledged to be a great advantage in the treatment of diseases in which the parenchyma is implicated. The instrument which the writer uses constantly is Pinkham's Improved Intra-Uterine Scarificator.* Dr. Miller's instrument,† as well as that of Dr. Storer,‡ in proper hands, leave nothing to be desired, although each of these gentlemen claims that his instrument is the *most* practical, which no doubt it is, in his own experienced hands.

The writer speaks highly of Pinkham's instrument, because he is familiar with it, and has succeeded by its means in verifying for himself the assertion of Dr. Storer: "I can truly say that I have repeatedly produced a greater amount of relief in a week or two, than I had previously been able to effect during many months."

*Pinkham's Intra-Uterine Scarificator.*

Here is the description of the instrument; it consists of two essential parts:

1st. A small knife attached to a slender staff; and

2d. A hollow tube, eleven inches in length, as a receptacle or sheath for the knife; this is furnished with a handle. This tube is curved and fashioned like a sound at its upper extremity. The concave surface of the upper part of the tube is perforated by an aperture $1\frac{1}{4}$ inch in length, through which the knife emerges when used for scarification. The extreme top of the tube is unscrewed whenever the instrument is cleaned, at which time the

* Journal of Gynæcological Society, Boston, Aug., 1869, upon Scarification of the Cavity of the Uterus, by J. G. Pinkham, Lynn, Mass.

† Boston Med. and Surg. Journal, March, 1867, p. 133.

‡ Transactions of the N. Y. Med. Society in 1867.

staff, to which the knife is attached, is pushed out of this aperture, and removed; it and the knife are then freed of blood.

The knife can in no instance emerge far enough to perforate even the walls of the thinnest uterus. Objections may be advanced against the use of this instrument on account of the injuries it might inflict. These objections, if they emanate from parties who have not used it, however respectable and eminent their position, are not entitled to any weight.

Intra-uterine scarification is a simple and safe operation in competent hands; only those, however, should attempt it who are adepts in the use of Emmet's uterine probe, not that serious damage of the parts can be inflicted, save contusions to the mucous membrane, but bungling produces pain.

The mode of performing intra-uterine scarification is as follows: The patient having been placed in the usual position, introduce Nott's speculum, ascertain by means of Emmet's probe the direction of the canal; follow the probe, on its withdrawal, by Pinkham's scarificator, scarify the uterus from fundus to cervix, in all directions before withdrawing it; this is succeeded by a probe armed with moistened cotton wool, to prevent the clogging of the cut surfaces with coagulated blood. After scarification apply a glycerine suppository, order hot vaginal injections, suppositories per rectum, etc.

It may, in conclusion, be proper to state that these scarifications remove in the aggregate a comparatively small quantity of blood, far less than leechings. It might else be inferred that these depletions react injuriously on the constitution; they must in all cases be limited to combat local congestions and these only.

The instruments described in this paper may be had of Messrs. George Tiemann & Co., of New York, and of their agents, Messrs. Bliss & Sharp, of this city.

169 Dearborn Street.

## Figures and Tables

**Figure f1:**
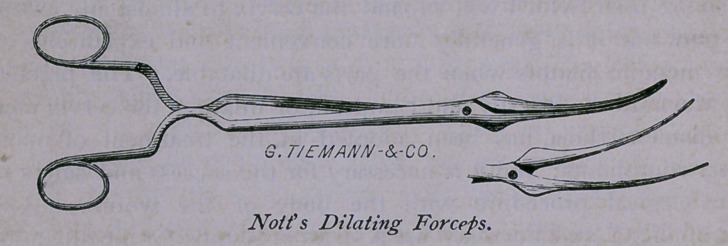


**Figure f2:**
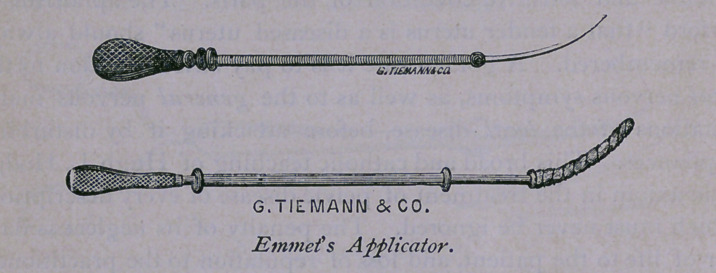


**Figure f3:**